# Change in weight and waist circumference and risk of colorectal cancer: results from the Melbourne Collaborative Cohort Study

**DOI:** 10.1186/s12885-016-2144-1

**Published:** 2016-02-25

**Authors:** Amalia Karahalios, Julie A. Simpson, Laura Baglietto, Robert J. MacInnis, Allison M. Hodge, Graham G. Giles, Dallas R. English

**Affiliations:** Centre for Epidemiology and Biostatistics, Melbourne School of Population and Global Health, The University of Melbourne, Bouverie Street, Melbourne, 3010 Australia; Cancer Epidemiology Centre, Cancer Council Victoria, 615 St Kilda Road, Melbourne, 3004 Australia; Team 9, Lifestyle, Genes and health: integrative trans-generational epidemiology, Inserm U1018, Centre for Research in Epidemiology and Population Health, Gustave Roussy Institute, 114 rue Edouard Vaillant, Villejuif Cedex, 94805 France; Paris-South University, Villejuif, France

**Keywords:** Anthropometry, Weight change, Waist circumference, Colorectal cancer, Prospective, Cohort

## Abstract

**Background:**

Studies reporting the association between change in weight or body mass index during midlife and risk of colorectal cancer have found inconsistent results, and only one study to date has reported the association between change in waist circumference (a measure of central adiposity) and risk of colorectal cancer.

**Methods:**

We investigated the association between risk of colorectal cancer and changes in directly measured waist circumference and weight from baseline (1990-1994) to wave 2 (2003-2007). Cox regression, with age as the time metric and follow-up starting at wave 2, adjusted for covariates selected from a causal model, was used to estimate the Hazard Ratios (HRs) and 95 % Confidence Intervals (CIs) for the change in waist circumference and weight in relation to risk of colorectal cancer.

**Results:**

A total of 373 cases of colorectal cancer were diagnosed during an average 9 years of follow-up of 20,605 participants. Increases in waist circumference and weight were not associated with the risk of colorectal cancer (HR per 5 cm increase in waist circumference = 1.02; 95 % CI: 0.95, 1.10; HR per 5 kg increase in weight = 0.93; 0.85, 1.02). For individuals with a waist circumference at baseline that was less than the sex-specific mean value there was a slight increased risk of colorectal cancer associated with a 5 cm increase in waist circumference at wave 2 (HR = 1.08; 0.97, 1.21).

**Conclusion:**

Increases in waist circumference and weight during midlife do not appear to be associated with the risk of colorectal cancer.

**Electronic supplementary material:**

The online version of this article (doi:10.1186/s12885-016-2144-1) contains supplementary material, which is available to authorized users.

## Background

There is substantial evidence that excess body fat, commonly measured by body mass index, increases the risk of colorectal cancer [1, 2]. Recently, interest has shifted to assessing whether adult weight gain also increases the risk [3]. Four recent systematic reviews and meta-analyses showed a positive association between weight change during adulthood and the risk of colorectal cancer [4–7].

Weight and body mass index might not be the best measures of the health risks associated with obesity since they provide no information on body fat content or distribution. Waist circumference and waist-to-hip ratio, simple measures of central or abdominal adiposity, have stronger associations with all-cause mortality, cardiovascular disease, cancer and type 2 diabetes compared with weight or body mass index [8–11]. To our knowledge, only one study assessed the association between prospective gain or loss in waist circumference during middle adult life and the risk of colorectal cancer [12].

Using a prospective cohort study in Melbourne, Australia, in which anthropometric measurements were directly measured at baseline and approximately 12 years later, we investigated associations between gain and loss in weight, waist and hips circumference during middle adult life and incidence of colorectal cancer.

## Methods

The Melbourne Collaborative Cohort Study is a prospective cohort study of 41,514 people (24,469 women), aged between 27 and 77 years at baseline (99.2 % of whom were aged 40 to 69 years). Participants were recruited between 1990 and 1994 (baseline) and attended clinics where demographic, anthropometric, lifestyle and dietary information were collected and anthropometric measurements were performed [13]. A follow-up clinic was conducted between 2003 and 2007 (wave 2) to update baseline information and repeat the anthropometric measurements. Participants gave written consent to participate in the study. Cancer Council Victoria’s Human Research Ethics Committee approved the study protocol.

### Exposure measures

All anthropometric measurements were taken by trained staff according to standard protocols. Height was measured at baseline, to 1 mm, using a stadiometer. At both baseline and wave 2, weight was measured to 100g using a digital electronic scale, and waist circumference and hips circumference were measured to 1 mm using a 2-meter metal anthropometric tape. Waist circumference was measured at the narrowest part of the torso and hips circumference was measured at the point of maximum circumference over the buttocks. For both waist circumference and hips circumference measurements participants were measured in light clothing with belts and restricting garments removed. Change in anthropometric measures were calculated as the value at baseline (1990–4) subtracted from the value at wave 2 (2003–7).

Information about country of birth and level of educational attainment was collected at baseline. Residential postcodes at baseline were used to classify participants into quintiles of an area-based measure of socioeconomic status [14]. At both waves, structured questionnaires were administered to collect information about physical activity, smoking status and diet [15]. A Mediterranean diet score, based on dietary and alcohol intake, was created at both waves of data collection. Smoking status was categorised as lifetime abstainer, quit before baseline, quit between baseline and wave 2, or current smoker at wave 2.

### Cohort follow-up and case ascertainment

Cases were participants with a primary diagnosis of adenocarcinoma of the colon or rectum (International Classification of Diseases, 10th revision: C18, C19 or C20) between date of wave 2 attendance and 30 June 2014. Cases were ascertained from record linkage to the population-based Victorian Cancer Registry and the Australian Cancer Database. Addresses and vital status of all participants were determined by record linkage to Electoral Rolls, Victorian death records, the National Death Index, from electronic phone books and from responses to mailed questionnaires and newsletters.

### Statistical analysis

Participants with extreme values for the baseline anthropometric variables (values below the 0.5 and above the 99.5 sex-specific percentiles of weight, waist and hips circumference, and of change in anthropometric measure) and energy intake were excluded due to potential measurement errors. Analyses for this paper were restricted to participants who attended both waves and who had not been diagnosed with any cancer before their wave 2 attendance.

The HRs for change in body size and the incidence of colorectal cancer were estimated using Cox regression with attained age as the time metric. Follow-up began on the date of the wave 2 measurement and ended at diagnosis of colorectal cancer (*n*=373), diagnosis of an unknown primary cancer (*n*=29), diagnosis of an in situ colorectal cancer or cancer of the anus (C21) (*n*=17), death (*n*=1814), or 30 June 2014 (*n*=18,362), whichever came first. To estimate separate HRs for colon (C18.0, 18.2 − 18.9) and rectal cancer (C19 and C20), we fitted competing risk models [16].

We used the likelihood ratio test to test the assumption of a linear association between the change in body size measures and the log(hazard) by comparing models with categorical (loss, stable, small gain and large gain) and pseudo-continuous change in body size variables. Because we did not find evidence of departure from linearity of associations for any of the anthropometric measures, we included them as continuous variables in the analyses. Tests based on Schoenfeld residuals showed no evidence that the proportional hazard assumptions were violated.

A causal diagram was developed and the following confounding variables were included in the models: country of birth, sex, quintile of socioeconomic status, family history of any cancer, the anthropometric measurement at baseline, cumulative smoking status, physical activity and Mediterranean diet score at baseline and wave 2 (Additional file 1) [17, 18].

We conducted sensitivity analyses to test whether the association between change in the anthropometric measures and incidence of colorectal cancer varied by sex, age at wave 2, body size at baseline (when participants were aged 40-69 years), smoking, length of time after wave 2, and undiagnosed diseases by fitting separate interaction terms between change in anthropometric measures and the following variables: (i) sex, (ii) age at wave 2 (≥ 65 vs < 65 years), (iii) baseline value of the anthropometric measure dichotomised at the sex-specific mean of body size (waist circumference: 94 cm for men and 80 cm for women; weight: 81 kg for men and 68 kg for women; hips circumference: 101 cm for men and 102 cm for women), (iv) smoking status (never smoked compared with ever smoked), (v) length of follow-up after wave 2 (first two years of follow-up compared with more than two years of follow-up), and (vi) previous history of disease (indicator for angina, diabetes or heart attack reported at baseline or wave 2), with the primary exposure of interest ‘the change in the anthropometric measure’ and tested the interactions with likelihood ratio tests.

Statistical analyses were performed using Stata version 13.1 [19].

## Results

**Fig. 1:**
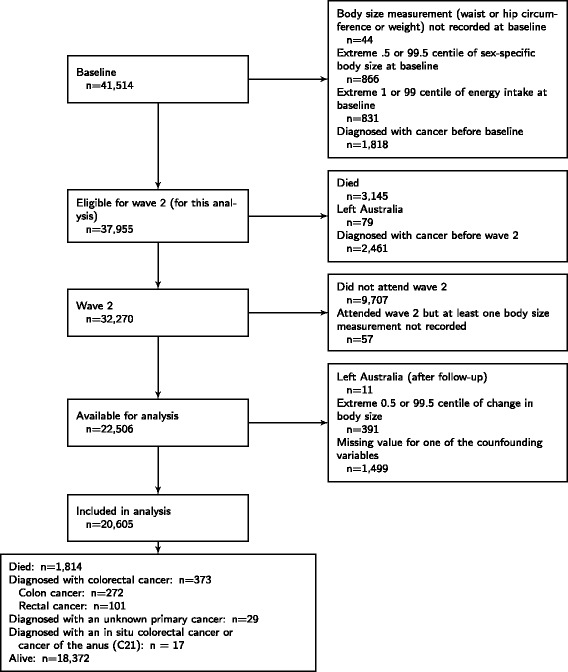
Flowchart of participants in the Melbourne Collaborative Cohort Study

Of the 41,514 participants in the Melbourne Collaborative Cohort Study, 44 did not have baseline anthropometric measurements, 866 had baseline measurements in the extreme 0.5 or 99.5 sex-specific centile, 831 had a total energy intake in the 1 or 99 centile at baseline, and 1818 had a diagnosis of cancer before baseline. Between baseline (1990–1994) and wave 2 (2003–2007), 3224 participants died or left Australia and 2,461 were diagnosed with cancer, leaving 32,270 available for invitation to wave 2 and eligible for this analysis. Of these participants, 9707 (30 %) did not attend wave 2, and 57 did not have at least one of their anthropometric measurements recorded (i.e. waist circumference, weight, or hips circumference) at wave 2. Finally, 1890 were excluded due to missing information for at least one of the confounding variables at baseline or wave 2, or for an extreme change in body size (i.e. 0.5 or 99.5 centile of sex-specific change in body size), leaving 20,605 (12,573 females) participants with complete data available for this analysis (Fig. 1).

Participants who attended wave 2 were more likely to have been born in Australia, New Zealand or the United Kingdom than Southern Europe, have attained a higher level of education, have never smoked, have low baseline alcohol intake, have a less disadvantaged socioeconomic status and be younger (Additional file 2). The mean baseline waist circumference, weight, and hips circumference for the participants included in the analysis were 83.6 cm, 72.3 kg, and 100.6 cm, respectively, and the mean changes in these measures were 7.0 cm, 2.2 kg, and 3.4 cm, respectively (Table 1 and Additional file 3). On average, the weight, waist and hips circumference increased from baseline to wave 2 (Table 1). About a third (34.7 %) of participants lost weight from baseline to wave 2, whereas only 15.9 % of participants decreased their waist circumference. The body size measurements at baseline and wave 2 were highly correlated (r for waist circumference = 0.82, r for weight = 0.91, and r for hips circumference = 0.76; Additional file 4).

**Table 1:** Distribution of body size measures at baseline and wave 2 for the Melbourne Collaborative Cohort Study participants

	All participants		Attended wave 2		
	Baseline			Baseline	Wave 2
	n	mean (SD)	n	mean (SD)	mean (SD)
Waist circumference (cm)					
All	41, 514	85.5 (13.0)	20, 595	83.6 (12.0)	90.5 (12.5)
Females	24, 469	80.0 (11.8)	12, 566	78.1 (10.5)	86.1 (11.9)
Males	17, 045	93.5 (10.0)	8029	92.1 (8.9)	97.5 (10.0)
Weight (kg)					
All	41, 514	73.4 (13.7)	20, 595	72.3 (12.7)	74.5 (13.6)
Females	24, 469	68.2 (12.4)	12, 566	67.1 (11.1)	69.7 (12.4)
Males	17, 045	80.8 (11.8)	8029	80.4 (10.7)	82.1 (11.9)
Hips circumference (cm)					
All	41, 514	101.4 (8.9)	20, 595	100.6 (7.9)	104.0 (8.9)
Females	24, 469	101.6 (10.0)	12, 566	100.7 (8.8)	104.5 (10.0)
Males	17, 045	101.1 (7.1)	8029	100.5 (6.2)	103.3 (6.6)

### Risk of colorectal cancer

There were 373 colorectal cancer cases (colon: 272 and rectal: 101) diagnosed over an average of 9.0 years after wave 2. Characteristics of the participants with and without colorectal cancer are shown in Additional file 5.

Table 2 shows the HRs of colorectal cancer for change in anthropometric measures from the following two models: a minimally adjusted model, included attained age during follow-up (as the time variable), sex, and country of birth (model 1), and a fully adjusted model, including the confounders in model 1, and additional confounders identified from a causal diagram (model 2). Results from model 2 show that increases in waist circumference, weight and hips circumference were not associated with an increased risk of colorectal cancer (for a 5 cm increase in waist circumference, HR = 1.02; 95 % CI: 0.95-1.10, 5kg increase in weight 0.93; 0.85-1.02), and a 5 cm increase in hips circumference, HR = 1.01; 0.92-1.10). We did not find evidence of departure from linearity of associations for any of the anthropometric measures (Additional file 6). There was little evidence of heterogeneity in the associations by subsite (Table 2).

**Table 2:** Incidence of colorectal cancer in relation to a 5 unit change in anthropometric measure: Hazard ratios and 95 % CI

	Model 1 ^*a*^			Model 2 ^*b*^		
	HR	95 % CI	*p*-value ^*c*^	HR	95 % CI	*p*-value ^*c*^
Colorectal (C18-20) ^*d*^						
Waist change (per 5 cm)	1.00	[0.93, 1.08]	0.924	1.02	[0.95, 1.10]	0.542
Weight change (per 5 kg)	0.92	[0.84, 1.02]	0.107	0.93	[0.85, 1.02]	0.147
Hips change (per 5 cm)	0.98	[0.90, 1.07]	0.654	1.01	[0.92, 1.10]	0.905
Colon (C18) ^*e*^						
Waist change (per 5 cm)	1.01	(0.93, 1.10)	0.777	1.03	(0.95, 1.12)	0.444
Weight change (per 5 kg)	0.94	(0.84, 1.05)	0.278	0.95	(0.86, 1.05)	0.327
Hips change (per 5 cm)	0.96	(0.87, 1.07)	0.484	0.99	(0.89, 1.10)	0.840
Rectal (C19,C20) ^*f*^						
Waist change (per 5 cm)	0.98	(0.86, 1.11)	0.763	1.00	(0.88, 1.13)	0.977
Weight change (per 5 kg)	0.88	(0.73, 1.06)	0.190	0.89	(0.75, 1.06)	0.191
Hips change (per 5 cm)	1.03	(0.88, 1.20)	0.747	1.05	(0.90, 1.22)	0.529

^*a*^Model 1: Estimates adjusted for sex and country of birth

^*b*^Model 2: Estimates adjusted as in model 1, as well as quintile of socioeconomic status, family history of any cancer, body size at baseline, cumulative smoking status, and physical activity and Mediterranean diet score at baseline and wave 2

^*c*^ *P*-values from Cox proportional hazard model

^*d*^373 colorectal cancer cases in 186,329 person-years at risk

Incidence rate of colorectal cancer = 2.00 per 1,000 person-years (95% CI = 1.81, 2.22)

^*e*^272 colon cancer cases (C18); Incidence rate = 1.46 per 1,000 person-years (95% CI = 1.30, 1.64)

^*f*^101 rectal cancer caases (C19,20); Incidence rate = 0.54 per 1,000 person-years (95% CI = 0.45, 0.66)

### Sensitivity analyses

The association between change in waist circumference and risk of colorectal cancer differed by the baseline value of waist circumference (*p*-value = 0.01 from likelihood ratio test). For individuals with baseline waist circumference below the sex-specific mean, the HR for an increase in waist circumference was slightly elevated (1.08 per 5 cm increase; 0.97-1.21), whereas it was not for those whose baseline waist circumference was above the sex-specific mean value: (0.97; 0.88-1.07) (Table 3). There was weak evidence that sex (*p*-value from likelihood ratio test: weight = 0.05, waist = 0.34, hips = 0.07) and previous history of disease (*p*-value from likelihood ratio test: weight = 0.05, waist = 0.61, hips = 0.03) modified the association (Additional file 7). Age at wave 2 (*p*-value from likelihood ratio test: weight = 0.13, waist = 0.78, hips = 0.37), smoking status (*p*-value from likelihood ratio test: weight = 0.16, waist = 0.67, hips = 0.38), and length of follow-up (*p*-value from likelihood ratio test: weight = 0.22, waist = 0.06, hips = 0.50) did not modify the associations for change in body size and risk of colorectal cancer.

**Table 3:** Risk of colorectal cancer in relation to 5 unit change in anthropometric measure by baseline value of the anthropometric measure: Hazard ratios and 95 % CI

	Baseline value of body size ^*a*^						
	<sex-specific mean value ^*d*^			≥sex-specific mean value ^*e*^			
	HR	95 % CI	*p*-value ^*b*^	HR	95 % CI	*p*-value ^*b*^	*p*-value ^*c*^
Waist change (per 5 cm)	1.08	[0.97, 1.21]	0.157	0.97	[0.88, 1.07]	0.502	0.01
Weight change (per 5 kg)	1.02	[0.88, 1.18]	0.826	0.88	[0.78, 0.99]	0.038	0.29
Hips change (per 5 cm)	1.04	[0.90, 1.20]	0.595	0.97	[0.86, 1.09]	0.583	0.14

^*a*^Sex-specific mean of baseline body size: Waist circumference: Males = 94 cm, Females = 80 cm; weight: Males = 81 kg, Females = 68 kg; Hips circumference: Males = 101 cm, Females = 102 cm

^*b*^ *P*-value from Cox proportional hazard model adjusted for sex, country of birth, family history of any cancer, quintile of socioeconomic status, baseline body size, cumulative smoking status and physical activity and Mediterranean diet score at baseline and wave 2

^*c*^ *P*-value from likelihood ratio test comparing the model with and without the interaction terms; where the interaction term is fitted between the covariate and the exposure of interest (i.e. change variable)

^*d*^176 colorectal cancer cases below the sex-specific mean value in 109,725 person-years at risk. Incidence rate = 1.60 per 1,000 person-years (95% CI = 1.38, 1.86)

^*e*^197 colorectal cancer cases above the sex-specific mean value in 76,605 person-years at risk. Incidence rate = 2.57 per 1,000 person-years (95% CI = 2.24, 2.96)

## Discussion

In this cohort study of middle-aged men and women, an increase of 5 units in waist circumference, weight or hips circumference, measured between 1990–1994 and approximately 12 years later, was not associated with a higher risk of colorectal cancer.

The strengths of our study include its prospective design, almost complete follow-up of participants after wave 2 (only 11 participants were known to have left Australia), updated covariate information at wave 2, and directly measured body size measurements, using standard protocols, at both waves.

Its principal limitations are the small number of colorectal cancer cases; attrition before wave 2 (approximately 30 % of participants alive at wave 2 did not attend the follow-up wave); and the lack of information on intentionality of weight change for the study participants.

The proportion of living participants attending wave 2 (i.e. 71.5 %) was similar to the proportion reported by other studies [20]. Those who attended both waves were younger, better educated, and had a healthier lifestyle than non-participants, which might restrict the findings to populations of fairly healthy middle-aged adults.

Prior to performing this analysis, we conducted an extensive simulation study to identify whether multiple imputation or complete-case analysis should be used to handle the missing anthropometric data at wave 2. We found that in the framework of this study, both methods provide unbiased estimates and there is minimal gain in precision when using multiple imputation [21]. Multiple imputation provides unbiased estimates when the data are ‘missing at random? Whether the missing data are ‘missing at random? or ‘missing not at random? is an untestable assumption. It has been suggested that for cohort studies which collect a large amount of information from their participants (as is this the case for the Melbourne Collaborative Cohort Study), the observed data can provide a large amount of information about the missing data. This is especially true for studies that invite participants to return to follow-up waves; where the baseline data are strongly predictive of the data at the follow-up waves (for example education status at baseline in our study which we control for in our Cox regression models).

Physical activity and diet (especially consumption of red and processed meat) may confound the association between obesity and risk of colorectal cancer [22]. A meta-analysis of 15 cohort studies suggested that the highest versus the lowest intake categories of red and processed meat were associated with 28 % and 21 % increased risk of colorectal cancer, respectively [23]. Information on red and processed meat intake was available at both waves of data collection; a Mediterranean diet score was calculated, giving lower scores for high meat intake and low fruit/vegetable consumption. Adjusting for Mediterranean diet score and physical activity at both waves did not materially change the findings.

To define strata of adiposity status, we used the mean sex-specific values for the participants of the Melbourne Collaborative Cohort Study (i.e. waist circumference = 94 cm for men and 80 cm for women, weight = 81 kg for men and 68 kg for women). These values correspond to the National Health and Medical Research Council Dietary Guidelines for Adults [24], which recommend maintaining a healthy weight with a waist circumference measurement less than 80 cm for women and 94 cm for men and a body mass index of between 18.5 and 25 kg/m ^2^. In our population, with an average height of 1.73 m for men and 1.61 m for women, a body mass index of 25 kg/m ^2^ corresponds to a weight of 75 kg for men and 65 kg for women.

Our results for weight gain showed a slight, non-statistically significant, decreased risk of colorectal cancer. Three recent meta-analyses showed that comparing the highest category of weight gain to a reference category was associated with an increased risk of colorectal cancer (HRs from 1.15 to 1.25) [4, 6, 7]. However, these pooled estimates incorporated weight change between early life and midlife, and weight change between midlife and older age. A meta-regression analysis showed that weight gain from early life to midlife was associated with a 1.23-fold increased risk of colorectal cancer (pooled HR = 1.23, 95 % CI = 1.14, 1.34) [4]. On the other hand, weight gain from midlife to older age was not associated with an increased risk of colorectal cancer (pooled HR = 1.02; 95 % CI = 0.91, 1.16) [4].

To date, only one study has looked at the association between change in waist circumference and the risk of colorectal cancer [12]. Song et al. relied on self-reported measurements of waist circumference and estimated the associations separately for men and women, using data from the Health Professionals Follow-up Study and the Nurses Health Study, respectively. A positive association was observed for men (HR for 10 cm gain = 1.34; 1.03, 1.74) but not for women (HR for 10 cm gain = 1.07; 0.93, 1.24). We did not find that sex modified the association between change in waist circumference and the risk of colorectal cancer.

Song et al. [12] also investigated the association between change in hips circumference and the risk of colorectal cancer. Similar to our results, they did not find an association between change in hips circumference and the risk of colorectal cancer (men: HR for 10 cm gain = 1.14; 0.93; 1.39; women: HR = 1.34; 0.99; 1.81).

We were unable to differentiate between unintentional and intentional weight change. As a result, reverse causation is a potential concern. When we excluded cancer cases diagnosed during the first two years of follow-up, the results were similar.

## Conclusions

In conclusion, we found no associations between changes in waist circumference, weight or hips circumference during middle adult life and the risk of colorectal cancer. However, previous studies have shown that weight gain from early life (i.e. age 18 to 21) to midlife is associated with an increased risk of colorectal cancer and weight gain from midlife to older age can have other detrimental effects. Therefore, recommendations should focus on maintaining a healthy body weight throughout the lifespan.

## Additional files

Additional file 1
**Causal diagram used to select additional confounding variables included in the analysis models.** (PDF 62 kb)

Additional file 2
**Distribution of baseline characteristics of the Melbourne Collaborative Cohort Study participants.** (PDF 93 kb)

Additional file 3
**Distribution of baseline demographic characteristics of the Melbourne Collaborative Cohort Study participants by change in anthropometric measure.** (PDF 61 kb)

Additional file 4
**Spearman rank correlations between body size measured at baseline and wave 2 and change in body size in the Melbourne Collaborative Cohort Study.** (PDF 31 kb)

Additional file 5
**Characteristics of the participants with and without colorectal cancer in the Melbourne Collaborative Cohort Study.** (PDF 63.4 kb)

Additional file 6
**Risk of colorectal cancer in relation to categories of change in anthropometric measures: Hazard ratios and 95 % CI.** (PDF 92.5 kb)

Additional file 7
**Risk of colorectal cancer in relation to a 5 unit change in anthropometric measures by sex and previous history of disease: Hazard ratios and 95 % CI.** (PDF 70.1 kb)

## Abbreviations

HR: hazard ratio
CI: confidence interval
