# Supplementary material for: Change in weight and waist circumference and risk of colorectal cancer: results from the Melbourne Collaborative Cohort Study
Source: BMC Cancer. 2016 Feb 25;16:157. doi: 10.1186/s12885-016-2144-1 (PMC4768408; doi:10.1186/s12885-016-2144-1)
Supplement: Additional file 1 — Causal diagram used to select additional confounding variables included in the analysis models. (PDF 62 kb) [file 12885_2016_2144_MOESM1_ESM.pdf]

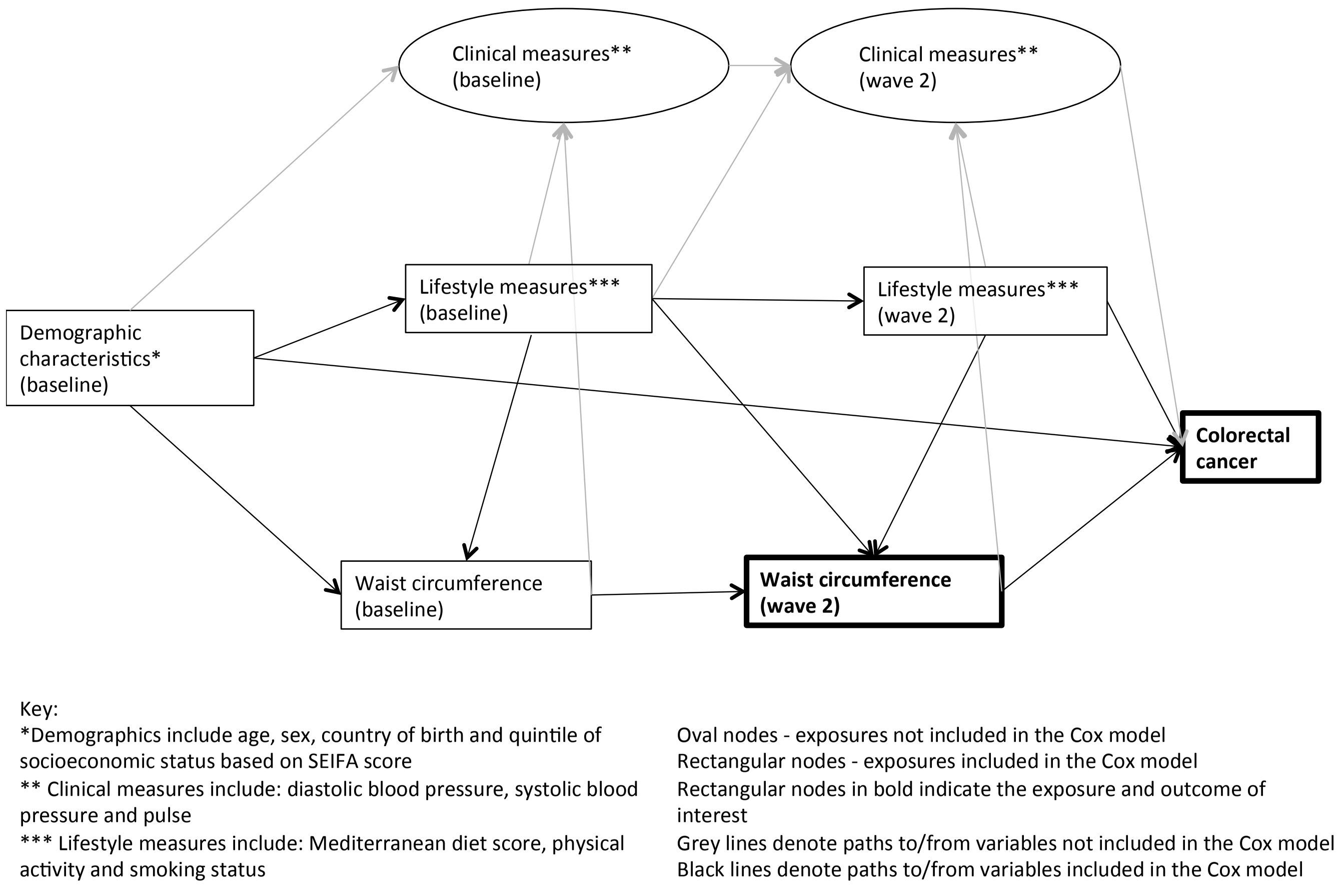

Additional file 1: Causal diagram used to select additional confounding variables included in the analysis models
