# Supplementary material for: Change in weight and waist circumference and risk of colorectal cancer: results from the Melbourne Collaborative Cohort Study
Source: BMC Cancer. 2016 Feb 25;16:157. doi: 10.1186/s12885-016-2144-1 (PMC4768408; doi:10.1186/s12885-016-2144-1)
Supplement: Additional file 2 — Distribution of baseline characteristics of the Melbourne Collaborative Cohort Study participants. (PDF 93 kb) [file 12885_2016_2144_MOESM2_ESM.pdf]

Additional file 2: Distribution of baseline characteristics of the Melbourne Collaborative Cohort Study participants

			Wave 2 attendance					
					Did not attend			
	Baseline		Attended		Alive		Died	
	n	%	n	%	n	%	n	%
**Participants**	41 514	100.0	26 984	65.0	10 731	25.8	3799	9.2
**Sex**								
Male	17 045	41.1	10 646	39.5	4217	39.3	2182	57.4
Female	24 469	58.9	16 338	60.5	6514	60.7	1617	42.6
**Age(years)**								
<50	13 152	31.7	9400	34.8	3465	32.3	287	7.6
50-59	13 507	32.5	9176	34.0	3508	32.7	823	21.7
≥60	14 855	35.8	8408	31.2	3758	35.0	2689	70.8
**Country of birth**								
Australia/New Zealand/UK	31 558	76.0	22 048	81.7	6727	62.7	2783	73.3
Southern Europe	9956	24.0	4936	18.3	4004	37.3	1016	26.7
**Highest level of education**								
Less than primary school	8046	19.4	3774	14.0	3274	30.5	998	26.3
Some high school	15 853	38.2	10 218	37.9	4123	38.4	1512	39.8
Completed high school	8576	20.7	5826	21.6	1962	18.3	788	20.8
Degree/diploma	9030	21.8	7164	26.6	1368	12.8	498	13.1
**Living alone**								
Not living alone	35 456	85.4	23 119	85.7	9260	86.3	3077	81.1
Living alone	6049	14.6	3863	14.3	1467	13.7	719	18.9
**Alcohol intake**[1]								
Lifetime abstainers	11 871	28.6	7005	26.0	3764	35.1	1102	29.1
Ex-drinkers	1663	4.0	963	3.6	462	4.3	238	6.3
Low intake	22 515	54.3	15 387	57.0	5272	49.2	1856	49.1
Moderate intake	3599	8.7	2507	9.3	735	6.9	357	9.4
High intake	1827	4.4	1113	4.1	484	4.5	230	6.1
**Mediterranean diet score**								
≤3	9797	23.6	6097	22.6	2661	24.8	1039	27.5
4	8725	21.0	5607	20.8	2306	21.5	812	21.5
5	9624	23.2	6286	23.3	2498	23.3	840	22.2
6	7887	19.0	5275	19.6	1947	18.2	665	17.6
≥7	5435	13.1	3708	13.7	1300	12.1	427	11.3
**Physical activity score**								
None - 0	9223	22.2	5529	20.5	2800	26.1	894	23.6
Low - >0 & <4	8326	20.1	5458	20.2	2167	20.2	701	18.5
Moderate - ≥4 & <6	14 776	35.6	9398	34.8	3803	35.5	1575	41.5
High - ≥6	9180	22.1	6597	24.4	1957	18.2	626	16.5
**Marital status**								

[1] Categories of alcohol intake are in grams/day as follows:
Low: Males: 1-39; Females: 1-19
Medium: Males: 40-59; Females: 20-39
High: Males: 60+; Females: 40+

	Wave 2 attendance							
					Did not attend			
	Baseline		Attended		Alive		Died	
	n	%	n	%	n	%	n	%
Married	28 500	71.8	18 718	71.9	7269	72.0	2513	70.4
Single	3615	9.1	2444	9.4	849	8.4	322	9.0
Divorced	3114	7.8	2056	7.9	825	8.2	233	6.5
de Facto	747	1.9	560	2.2	164	1.6	23	0.6
Widow	2801	7.1	1666	6.4	731	7.2	404	11.3
Separated	914	2.3	576	2.2	264	2.6	74	2.1
**Smoking status**								
Never smokers	23 819	57.4	16 212	60.1	5991	55.8	1616	42.6
Former smokers	12 997	31.3	8328	30.9	3162	29.5	1507	39.7
Current smokers	4688	11.3	2441	9.0	1574	14.7	673	17.7
**Index of Relative Socioeconomic Disadvantage**								
1st Quintile (most disadvantaged)	6083	14.7	3476	12.9	1918	17.9	689	18.2
2nd Quintile	8620	20.8	5014	18.6	2653	24.8	953	25.2
3rd Quintile	7668	18.5	4801	17.8	2116	19.8	751	19.8
4th Quintile	8447	20.4	5818	21.6	1975	18.5	654	17.3
5th Quintile (least disadvantaged)	10 559	25.5	7798	29.0	2024	18.9	737	19.5
