# Supplementary material for: Change in weight and waist circumference and risk of colorectal cancer: results from the Melbourne Collaborative Cohort Study
Source: BMC Cancer. 2016 Feb 25;16:157. doi: 10.1186/s12885-016-2144-1 (PMC4768408; doi:10.1186/s12885-016-2144-1)
Supplement: Additional file 3 — Distribution of baseline demographic characteristics of the Melbourne Collaborative Cohort Study participants by change in anthropometric measure. (PDF 61 kb) [file 12885_2016_2144_MOESM3_ESM.pdf]

Additional file 3: Distribution of baseline characteristics of the Melbourne Collaborative Cohort Study participants by change in anthropometric measure

| | Weight (kg) | | Waist (cm) | | Hips (cm) | |
|---|---|---|---|---|---|---|
| | Mean | SD | Mean | SD | Mean | SD |
| All participants | 7.0 | 7.3 | 2.2 | 5.8 | 3.4 | 5.9 |
| **Sex** | | | | | | |
| Male | 5.4 | 6.6 | 1.7 | 5.4 | 2.8 | 5.0 |
| Female | 8.0 | 7.5 | 2.5 | 6.0 | 3.8 | 6.3 |
| **Country of birth** | | | | | | |
| Australia/New Zealand/UK | 6.9 | 7.3 | 2.2 | 5.8 | 3.4 | 5.8 |
| Southern Europ | 7.2 | 7.5 | 2.5 | 5.8 | 3.6 | 6.0 |
| **Quintiles of socio-economic disadvantage** | | | | | | |
| 1st quintile (most disadvantaged) | 7.4 | 7.8 | 2.3 | 6.2 | 3.7 | 6.4 |
| 2nd quintile | 7.1 | 7.6 | 2.4 | 6.1 | 3.6 | 6.1 |
| 3rd quintile | 7.0 | 7.3 | 2.3 | 5.8 | 3.5 | 5.9 |
| 4th quintile | 7.0 | 7.2 | 2.3 | 5.7 | 3.5 | 5.8 |
| 5th quintile (least disadvantaged) | 6.7 | 7.0 | 1.9 | 5.4 | 3.1 | 5.5 |
| **Physical activity score** | | | | | | |
| None - 0 | 7.3 | 7.6 | 2.4 | 6.1 | 3.7 | 6.2 |
| Low - >0 and <4 | 7.0 | 7.1 | 2.2 | 5.7 | 3.4 | 5.9 |
| Moderate - ≥4 and <6 | 7.0 | 7.3 | 2.0 | 5.8 | 3.4 | 5.8 |
| High - ≥6 | 6.8 | 7.1 | 2.3 | 5.4 | 3.3 | 5.6 |
| **Mediterranean diet score** | | | | | | |
| ≤3 | 7.0 | 7.5 | 2.3 | 6.1 | 3.4 | 6.0 |
| 4 | 7.1 | 7.4 | 2.3 | 5.9 | 3.5 | 5.9 |
| 5 | 7.0 | 7.3 | 2.2 | 5.8 | 3.4 | 5.9 |
| 6 | 6.9 | 7.2 | 2.1 | 5.5 | 3.3 | 5.8 |
| ≥7 | 6.8 | 6.9 | 2.0 | 5.5 | 3.2 | 5.5 |
| **Family history of any cancer** | | | | | | |
| No | 7.0 | 7.3 | 2.3 | 5.8 | 3.5 | 5.8 |
| Yes | 6.9 | 7.3 | 2.0 | 5.8 | 3.3 | 5.9 |
| **Cumulative smoking status** | | | | | | |
| Lifetime abstainer | 7.0 | 7.3 | 2.2 | 5.6 | 3.5 | 5.9 |
| Quit before baseline | 6.5 | 7.2 | 1.9 | 5.7 | 3.1 | 5.7 |
| Quit between baseline and follow-up | 9.7 | 7.3 | 4.9 | 6.2 | 5.0 | 5.9 |
| Current | 6.8 | 7.7 | 2.0 | 6.4 | 2.9 | 6.0 |
| **Physical activity score at follow-up** | | | | | | |
| None - 0 | 7.2 | 8.0 | 2.2 | 6.9 | 3.8 | 6.5 |
| Low - >0 and <4 | 8.0 | 7.4 | 3.0 | 6.3 | 4.4 | 6.3 |
| Moderate - ≥4 and <6 | 7.2 | 7.5 | 2.3 | 5.9 | 3.5 | 5.9 |
| High - ≥6 | 6.7 | 7.1 | 2.1 | 5.5 | 3.2 | 5.7 |
| **Mediterranean diet score at follow-up** | | | | | | |
| ≤3 | 7.1 | 7.4 | 2.4 | 6.1 | 3.6 | 5.9 |
| 4 | 7.2 | 7.3 | 2.4 | 5.8 | 3.4 | 6.0 |
| 5 | 7.0 | 7.3 | 2.2 | 5.6 | 3.4 | 5.8 |
| 6 | 6.9 | 7.2 | 2.1 | 5.6 | 3.4 | 5.8 |
| ≥7 | 6.5 | 7.1 | 1.8 | 5.4 | 3.0 | 5.6 |
