# Supplementary material for: Change in weight and waist circumference and risk of colorectal cancer: results from the Melbourne Collaborative Cohort Study
Source: BMC Cancer. 2016 Feb 25;16:157. doi: 10.1186/s12885-016-2144-1 (PMC4768408; doi:10.1186/s12885-016-2144-1)
Supplement: Additional file 4 — Spearman rank correlations between body size measured at baseline and wave 2 and change in body size in the Melbourne Collaborative Cohort Study. (PDF 31 kb) [file 12885_2016_2144_MOESM4_ESM.pdf]

Additional file 4: Spearman rank correlations between body size measured at baseline and wave 2 and change in body size in the Melbourne Collaborative Cohort Study

		Baseline			Wave 2			Absolute change		
		Waist (cm)	Weight (kg)	Hips (cm)	Waist (cm)	Weight (kg)	Hips (cm)	Waist (cm)	Weight (kg)	Hips (cm)
Baseline	Waist(cm)	1.00								
	Weight(kg)	0.85	1.00							
	Hips(cm)	0.65	0.70	1.00						
Wave 2	Waist(cm)	0.82	0.80	0.59	1.00					
	Weight(kg)	0.75	0.91	0.64	0.86	1.00				
	Hips(cm)	0.50	0.62	0.76	0.71	0.74	1.00			
Absolute change	Waist(cm)	-0.24	-0.03	-0.05	-	-	-	1.00		
	Weight(kg)	-0.10	-0.05	-0.02	-	-	-	0.64	1.00	
	Hips(cm)	-0.12	-0.01	-0.20	-	-	-	0.65	0.61	1.00
