# Supplementary material for: Change in weight and waist circumference and risk of colorectal cancer: results from the Melbourne Collaborative Cohort Study
Source: BMC Cancer. 2016 Feb 25;16:157. doi: 10.1186/s12885-016-2144-1 (PMC4768408; doi:10.1186/s12885-016-2144-1)
Supplement: Additional file 5 — Characteristics of the participants with and without colorectal cancer in the Melbourne Collaborative Cohort Study. (PDF 63.4 kb) [file 12885_2016_2144_MOESM5_ESM.pdf]

Additional file 5: Characteristics of the participants with and without colorectal cancer in the Melbourne Collaborative Cohort Study

	Non-cases n(%) or mean[SD]	Cases n(%) or mean[SD]
**Participants**	20,232 (100.0)	373 (100.0)
**Sex**		
Male	7,849 (38.8)	183 (49.1)
Female	12,383 (61.2)	190 (50.9)
**Country of birth**		
Australia/New Zealand/UK	17,077 (84.4)	317 (85.0)
Southern Europe	3,155 (15.6)	56 (15.0)
**Index of Relative Socioeconomic Disadvantage**		
1st Quintile (most disadvantaged)	2,483 (12.3)	38 (10.2)
2nd Quintile	3,605 (17.8)	81 (21.7)
3rd Quintile	3,575 (17.7)	62 (16.6)
4th Quintile	4,473 (22.1)	89 (23.9)
5th Quintile (least disadvantaged)	6,096 (30.1)	103 (27.6)
**Highest level of education**		
Less than primary school	2,310 (11.4)	46 (12.3)
Some high school	7,706 (38.1)	148 (39.7)
Completed high school	4,459 (22.0)	103 (27.6)
Degree/diploma	5,757 (28.5)	76 (20.4)
**Alcohol intake**		
Lifetime abstainers	5,073 (25.1)	86 (23.1)
Ex-drinkers	669 (3.3)	16 (4.3)
Low intake	11,778 (58.2)	201 (53.9)
Moderate intake	1,901 (9.4)	39 (10.5)
High intake	811 (4.0)	31 (8.3)
**Mediterranean diet score**		
$\leq$3	4,503 (22.3)	86 (23.1)
4	4,210 (20.8)	89 (23.9)
5	4,741 (23.4)	86 (23.1)
6	3,982 (19.7)	58 (15.5)
$\geq$7	2,796 (13.8)	54 (14.5)
**Physical activity score**		
None - 0	3,973 (19.6)	84 (22.5)
Low - $>$0 & $<$4	4,074 (20.1)	85 (22.8)
Moderate - $\geq$4 & $<$6	6,954 (34.4)	129 (34.6)
High - $\geq$6	5,231 (25.9)	75 (20.1)
**Marital status**		
Married	14,109 (71.9)	265 (72.4)
Single	1,831 (9.3)	31 (8.5)
Divorced	1,559 (7.9)	30 (8.2)
de Facto	457 (2.3)	4 (1.1)
Widow	1,212 (6.2)	30 (8.2)
Separated	457 (2.3)	6 (1.6)

	Non-cases n(%) or mean[SD]	Cases n(%) or mean[SD]
**Smoking status (cumulative smoking status)**		
Lifetime abstainer	12,371 (61.1)	211 (56.6)
Quit before baseline	5,961 (29.5)	130 (34.9)
Quit between baseline and follow-up	881 (4.4)	18 (4.8)
Current	1,019 (5.0)	14 (3.8)
**Physical activity score at follow-up**		
None (0)	351 (1.7)	4 (1.1)
Low (0,4)	1,763 (8.7)	41 (11.0)
Moderate [4,6)	6,195 (30.6)	123 (33.0)
High [6,16)	11,923 (58.9)	205 (55.0)
**Mediterranean diet score at follow-up**		
$\leq$3	5,418 (26.8)	113 (30.3)
4	4,300 (21.3)	91 (24.4)
5	4,549 (22.5)	70 (18.8)
6	3,504 (17.3)	53 (14.2)
$\geq$7	2,461 (12.2)	46 (12.3)
**Hips change (cm)**	3.4 [5.9]	2.9 [5.6]
**Waist change (cm)**	7.0 [7.3]	6.3 [7.0]
**Weight change (kg)**	2.2 [5.8]	0.9 [5.5]
