# Supplementary material for: Change in weight and waist circumference and risk of colorectal cancer: results from the Melbourne Collaborative Cohort Study
Source: BMC Cancer. 2016 Feb 25;16:157. doi: 10.1186/s12885-016-2144-1 (PMC4768408; doi:10.1186/s12885-016-2144-1)
Supplement: Additional file 6 — Risk of colorectal cancer in relation to categories of change in anthropometric measures: Hazard ratios and 95 % CI. (PDF 92.5 kb) [file 12885_2016_2144_MOESM6_ESM.pdf]

Additional file 6: Risk of colorectal cancer in relation to categories of change in anthropometric measures: Hazard Ratio and 95% CI

					Model 1[a]			Model 2[b]	
	Deaths	Person-years	Rate[c]	HR	95% CI	p-value	HR	95% CI	p-value
**Waist change (cm)**									
Loss (≤-5 cm)	17	8,636	1.97	0.78	[0.47, 1.28]	0.321	0.72	[0.43, 1.19]	0.197
Stable (±5 cm)	155	68,288	2.27	1.00	-	-	1.00	-	-
Small gain (>5 and ≤10 cm)	96	50,714	1.89	0.90	[0.70, 1.16]	0.405	0.92	[0.72, 1.19]	0.545
Large gain (>10 cm)	105	58,691	1.79	0.95	[0.74, 1.22]	0.693	0.99	[0.77, 1.28]	0.952
**Weight change (kg)**									
Loss (≤-3 kg)	87	28,830	3.02	1.20	[0.92, 1.56]	0.175	1.10	[0.84, 1.43]	0.493
Stable (±3 kg)	164	78,617	2.09	1.00	-	-	1.00	-	-
Small gain (>3 and ≤8 kg)	80	51,787	1.54	0.89	[0.68, 1.17]	0.400	0.87	[0.66, 1.14]	0.306
Large gain (>8 kg)	42	27,096	1.55	1.06	[0.75, 1.50]	0.743	0.97	[0.68, 1.37]	0.858
**Hips change (cm)**									
Loss (≤-3 cm)	46	23,091	1.99	0.87	[0.62, 1.21]	0.403	0.80	[0.57, 1.12]	0.187
Stable (±3 cm)	156	70,883	2.20	1.00	-	-	1.00	-	-
Small gain (>3 and ≤8 cm)	119	56,730	2.10	1.03	[0.81, 1.30]	0.839	1.05	[0.83, 1.34]	0.672
Large gain (>8 cm)	52	35,625	1.46	0.78	[0.57, 1.07]	0.125	0.81	[0.59, 1.11]	0.186

[a] Model 1: Estimates adjusted for sex and country of birth.

[b] Model 2: Estimates adjusted for sex, country of birth, quintile of socioeconomic status, body size at baseline, cumulative smoking status, and the following covariates measured at baseline and wave 2: physical activity, Mediterranean diet score and living alone.

[c] Rate per 1,000 person-years
