# Supplementary material for: Change in weight and waist circumference and risk of colorectal cancer: results from the Melbourne Collaborative Cohort Study
Source: BMC Cancer. 2016 Feb 25;16:157. doi: 10.1186/s12885-016-2144-1 (PMC4768408; doi:10.1186/s12885-016-2144-1)
Supplement: Additional file 7 — Risk of colorectal cancer in relation to a 5 unit change in anthropometric measures by sex and previous history of disease: Hazard ratios and 95 % CI. (PDF 70.1 kb) [file 12885_2016_2144_MOESM7_ESM.pdf]

Additional file 7: Risk of colorectal cancer in relation to 5 unit change in anthropometric measure by sex and previous history of disease: Hazard ratios and 95% CI

	Cases (Person-years)	HR	95% CI	p-value	Cases (Person-years)	HR	95% CI	p-value
	**Males**				**Females**			
Hips change (per 5cm)	183 (71,645)	1.02	[0.99, 1.05]	0.235	190 (114684)	0.99	[0.97, 1.02]	0.510
Waist change (per 5cm)	183 (71,645)	1.01	[0.99, 1.04]	0.287	190 (114684)	1.00	[0.98, 1.02]	0.954
Weight change (per 5kg)	183 (71,645)	1.00	[0.97, 1.03]	0.869	190 (114684)	0.98	[0.95, 1.00]	0.085
	**No history of disease**				**History of disease**			
Hips change (per 5cm)	133 (54,571)	0.99	[0.97, 1.01]	0.293	190 (114684)	1.02	[0.99, 1.05]	0.304
Waist change (per 5cm)	133 (54,571)	1.01	[0.99, 1.03]	0.465	190 (114684)	1.00	[0.98, 1.02]	0.996
Weight change (per 5kg)	133 (54,571)	0.98	[0.95, 1.00]	0.039	190 (114684)	1.00	[0.97, 1.03]	0.805

[a] P-value from Cox proportional hazard model adjusted for sex, country of birth, family history of any cancer, quintile of socioeconomic status, baseline body size, cumulative smoking status and physical activity and Mediterranean diet score at baseline and wave 2.
